# Intravoxel Incoherent Motion Magnetic Resonance Imaging with Integrated Slice-specific Shimming for old myocardial infarction: A Pilot Study

**DOI:** 10.1038/s41598-019-56489-6

**Published:** 2019-12-24

**Authors:** Shi-Feng Xiang, Xue-Qiang Zhang, Su-Jun Yang, Yun-Yun Gao, Bu-Lang Gao, Qing-Lei Shi, Shuai Li

**Affiliations:** 1 Handan Central Hospital, 15 Southern Zhonghua Street, Handan City, Hebei Province 056001 China; 2 Siemens medical system Co. Ltd., Beijing, 100176 China

**Keywords:** Magnetic resonance imaging, Ischaemia

## Abstract

Currently, little is known regarding the value of quantitative parameters derived from the intravoxel incoherent motion (IVIM) magnetic resonance imaging (MRI) with integrated slice-specific shimming (iShim) sequence in detecting old myocardial infarction and myocardial fibrosis. This study was to investigate the value of IVIM-MRI with iShim sequence in diagnosing old myocardial infarction and fibrosis. Thirty-five patients with both old myocardial infarction and myocardial fibrosis and 12 healthy volunteers were prospectively enrolled to undergo cardiac diffusion-weighted imaging (DWI) using seven b-values (0, 20, 60, 80, 120, 200 and 600 s/mm^2^). The iShim sequence was used for IVIM data acquisition, and the diffusion parameters, D, D* and *f* values for IVIM, and conventional apparent diffusion coefficient (ADC) were evaluated on the anterior, posterior and lateral walls of the ventricular septum using the short axis of the heart. Significant differences were found in the D, D* and *f* values between healthy subjects and patients with old myocardial infarction and myocardial fibrosis (*P* = 0.000), with the median value of the D and *f* significantly smaller in the myocardial infarction and fibrosis than in the normal control but the median value of D* significantly greater in the myocardial infarction and fibrosis than in the normal control. In the receiver operating curve analysis, the areas under the curve were 0.939, 0.988 and 0.959 for the D, D* and *f* values, respectively. The sensitivities and specificities were 84.6% and 94.4% for D, 88.9% and 84.6% for D* and 100% and 93.1% for the *f* values, respectively. In conclusion, **t**he IVIM-derived parameters (D, D* and *f*) obtained using the iShim DWI technique showed high capacity in diagnosing old myocardial infarction and myocardial fibrosis by providing diffusion and perfusion information, which may have great importance in future clinical practice.

## Introduction

Myocardial infarction is one of the leading causes of death and disability worldwide, and its incidence in China is increasing annually. Myocardial fibrosis at the sites of old myocardial infarction is a determining factor of ventricular remodelling, which changes the mechanical and electrical activities of the heart and eventually leads to heart failure and death in severe cases. Thus, detection of myocardial fibrosis plays an important role in clinical treatment strategies. In recent decades, late gadolinium enhancement (LGE) in cardiac magnetic resonance (CMR) has become the gold standard for evaluating myocardial infarction and localised fibrosis^1,2^. However, a considerable number of patients cannot undergo enhanced cardiac scanning because of contrast agent allergy, renal fibrosis or other reasons.

Diffusion-weighted imaging (DWI) is a quantitative detection method that can detect the diffusion of water molecules in the tissues; it is an imaging technique widely applied in many whole-body systems in clinical practice. However, the apparent diffusion coefficient (ADC) in traditional DWI is calculated using a single-exponential model based on the premise that the diffusion motion of water molecules in tissues follows a single Gaussian distribution, ignoring the effect of perfusion on the expansion motion. Moreover, myocardial ischaemic injury is generally caused by changes in myocardial perfusion. Thus, accurate assessment of myocardial diffusion and perfusion abnormalities is valuable in the diagnosis of cardiac ischaemic diseases.

The intravoxel incoherent motion (IVIM) theory, proposed by Le Bihan *et al*.^3^, enables evaluation of living tissue diffusion movement and micro-vessel perfusion *in vivo* using quantitative parameters obtained from the multi-b-value DWI of a double-exponential decay model. At present, the proposed theory has been widely used in studies of the head and neck^4^, liver^5^ and vertebral body^6^, with a relatively high diagnostic value. IVIM can reflect the diffusion and perfusion of living tissues through multi-parameter measurements. Therefore, IVIM may play an important supplementary role to conventional CMR in patients with heart disease who cannot tolerate drug therapy^7,8^. We hypothesized that the IVIM-DWI had a good value in diagnosing old myocardial infarction and myocardial fibrosis. This study consequently was performed to investigate the value of the diffusion coefficient (D value), perfusion coefficient (D* value) and perfusion fraction (*f* value) measured by the IVIM-DWI in diagnosing old myocardial infarction and myocardial fibrosis in comparison with healthy volunteers.

## Patients and Methods

### Patients

Thirty-five patients with both old myocardial infarction and fibrosis were prospectively enrolled between May 2016 and September 2018, comprising 28 males and 7 females aged 36–75 years with a mean age of 53.6 years. Another 12 healthy volunteers were also prospectively enrolled as the controls, comprising seven males and five females aged 23–53 years with a mean age of 35.7 years. The inclusion criteria were as follows: ① for patients, a definite medical history of myocardial infarction with duration of infarction ≥6 months; ② for healthy volunteers, no previous history of cardiopulmonary or cerebrovascular diseases and ③ for all subjects, multi-b-value DWI and contrast-enhanced magnetic resonance imaging (CE-MRI) were performed. The exclusion criteria were as follows: ① patients with severe arrhythmia or those failing to cooperate with the study and ② patients who could not undergo MRI examination (because of claustrophobia, implantation of pacemakers or defibrillators, etc). This study was approved by the Institutional Review Board of Handan Central Hospital, and written informed consent was obtained from all subjects including the healthy volunteers. All methods were performed in accordance with the relevant guidelines and regulations.

### MRI acquisition

Imaging was performed with a 3.0-T MRI scanner (Magnetom Skyra 3.0 T, Siemens Healthcare, Erlangen, Germany) with a phased array surface coil. Respiration navigator and electrocardiogram (ECG) triggering technique were used in the course of the scanning. The planes included in the cardiac morphology examination included the routine two-chamber plane, four-chamber plane and a series of short-axis cine. Myocardial perfusion and delayed myocardial enhancement were performed in all the subjects. The IVIM-DWI was performed on the short axis of the heart with b-values of 0, 20, 60, 100, 150, 200 and 600 s/mm^2^. The scanning parameters were as follows: frequency-coded field-of-view of 306 mm, phase-coded field-of-view of 75%, repetition time of 2,200.0 ms, echo time of 67 ms, layer thickness of 8 mm, scanning interval of 1.5–3.5 mm, number of excitation as 8.00 times, with local shimming, respiration navigator and ECG triggering technique integrated during the acquisition. Regarding directions of diffusion gradients, a three-scan trace diffusion gradient scheme was applied. In this scheme, the directions of diffusion gradient are orthogonal and line up with the directions of x, y and z axis.

### MRI analysis

The LGE-positive segments of old myocardial infarction were analysed by two radiologists with 6 and 10 years of experience, respectively, in cardiovascular imaging diagnosis. The IVIM parameter maps were reconstructed using a prototype software (Body Diffusion Toolbox, Siemens Healthcare). In this experiment, a more numerically-stable ‘segmented’ analysis procedure was performed, which is commonly used by most researchers to calculate the relevant parameters. In this method, because the value of D* is significantly greater than that of D, the b-value is significantly greater than ~1/D* (e.g. for D* ~10 µm^2^/ms, b > 100 s/mm^2^) and the influence of the pseudo-diffusion term on the signal decay is quite small. Thus, in this higher b-value regime, the bi-exponential model can be simplified into an exponential fit equation:

$${{\rm{S}}}_{{\rm{b}}}={{\rm{S}}}_{0}((1-{\rm{f}})\,\exp \,(-\,{\rm{b}}\ast {\rm{D}}))$$

Operationally, D value is determined from such a mono-exponential fit to data above a chosen threshold (in this study, b > 170 s/mm^2^), whose zero intercept Sint is used along with the unweighted (b = 0) signal S0 to determine the *f* value, according to the following formula:

$$f=({S}_{0}-{S}_{int})/{S}_{0}$$

Thus, finally, D* was calculated from the measured D and *f* values, in accordance with the partially-constrained nonlinear fit method, using the bi-exponential model. In the case of parametric map analysis, an additional step was included (residual masking) to avoid overfitting the mono-exponential voxels. Subsequently, the parameter maps obtained for IVIM were transferred into a Syngo via workstation for measurement. To locate the infarcted myocardium with enhanced accuracy, short-axis delayed-enhancement positive images were selected in the delineated region of interest of the infarcted myocardium. Meanwhile, normal myocardium was evaluated at the anterior, posterior and lateral walls of the ventricular septum. Eventually, the D, D* and *f* values were obtained, and all the parameter values were expressed as mean ± standard deviation.

### Statistical analysis

The SPSS19.0 statistical software (IBM SPSS Statistics for Windows, Version 19.0. Armonk, NY, USA) was used for the analysis of the experimental data. Measurement data were expressed as mean ± SD (standard deviation). The rank sum test was performed for the D, D* and *f* values of the myocardium, in the old myocardial infarction of the patients and the normal myocardium of the healthy volunteers. Furthermore, the diagnostic value for the fibrosis of myocardial infarction was evaluated by receiver operating characteristic (ROC) curve analysis for the D, D* and *f* values, followed by the calculation of accuracy, sensitivity, specificity and the positive and negative predictive values in diagnosing the fibrosis of myocardial infarction. A *P* < 0.05 was considered to be statistically significant.

## Results

### General characteristics of IVIM-DWI

**Figure 1:**
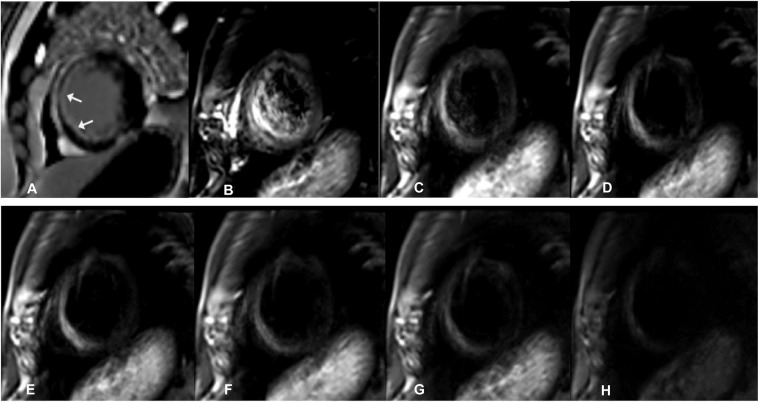
A male patient aged 56 years with old myocardial infarction. (**A**) Magnetic resonance imaging (MRI) with delayed enhancement was performed with LGE (late gadolinium enhancement)-positive area of infarction indicated by the arrows. B-H. IVIM-DWI images of the same patient. The IVIM-DWI images were shown with b values of 0 (**B**), 20 (**C**), 60 (**D**), 80 (**E**), 120 (**F**), 200 (**G**), and 600 (**G**) s/mm^2^. Both the signal intensity of the infarcted myocardium and cardiac blood pool demonstrated high signal intensity. With the increase in the b value, the signal intensity of the blood pool decreased, with an optimal contrast at b = 120 s/mm^2^. IVIM, intravoxel incoherent motion; DWI, diffusion-weighted image.

**Figure 2:**
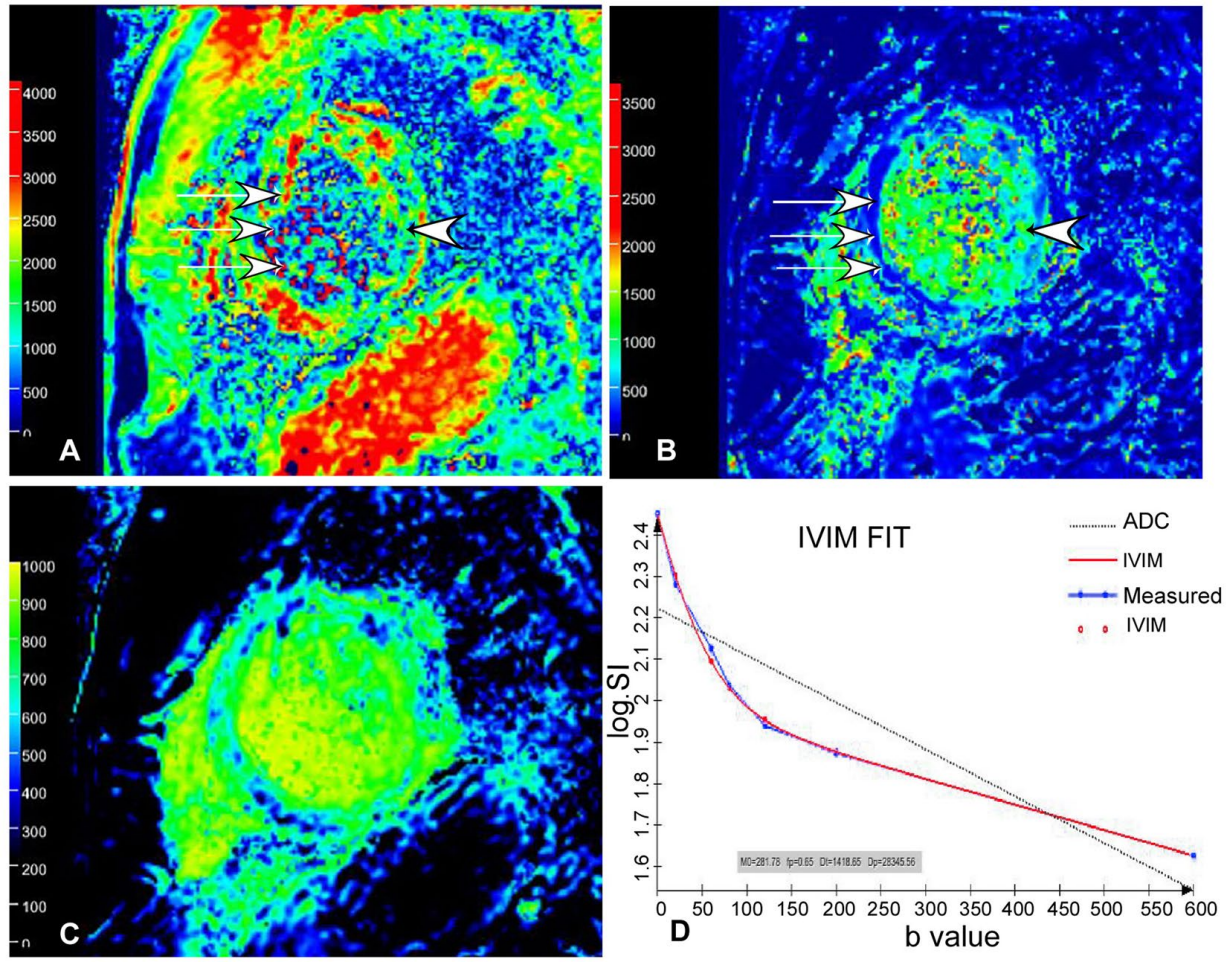
Pseudo-color images of D (**A**), D* (**B**) and ***f*** (**C**) value in IVIM-DWI images in the same 56-year-old man as in Fig. 1. (**A**) The D value of the infarction (white arrows) of the interventricular septum was significantly lower than that (arrow head) of the normal lateral wall myocardium. (**B**) The D* value of the infarction (white arrows) of the interventricular septum was significantly greater than that (arrow head) of the normal lateral wall myocardium. (**C**) The ***f*** value of the infarction of the interventricular septum was significantly greater than that of the normal lateral wall myocardium. The D, D* and ***f*** values of the infarction of the interventricular septum were 0.0016 × 10^−3^ mm^2^/s, 0.79 × 10^−3^ mm^2^/s and 0.14, respectively. (**D**) In the fitting curves, the measured signal intensity points are distributed near the fitted curves, which reflect a higher image quality without obvious impact by respiratory movement and heart beat. Meanwhile, the dual-exponential algorithm demonstrates a better fitting effect than mono-exponential algorithm. D value, diffusion coefficient; D* value, perfusion coefficient; ***f*** value, perfusion fraction; IVIM, intravoxel incoherent motion; DWI, diffusion-weighted image.

**Figure 3:**
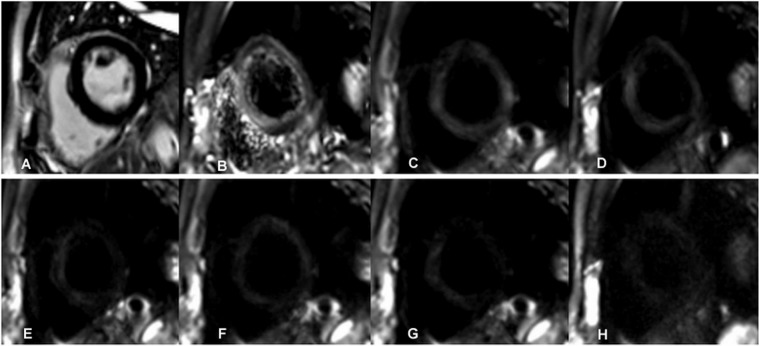
Images of a 51-year-old healthy volunteer were shown. (**A**) No abnormal signal was detected in the late gadolinium enhancement (LGE) images. (**B**–**H**) Diffusion weighted images had the b value of 0 (**B**), 20 (**C**), 60 (**D**), 80 (**E**), 120 (**F**), 200 (**G**), 600 (**H**) s/mm^2^ respectively. The myocardial signal decreased with increase of the b values. The myocardium had a uniform signal.

**Figure 4:**
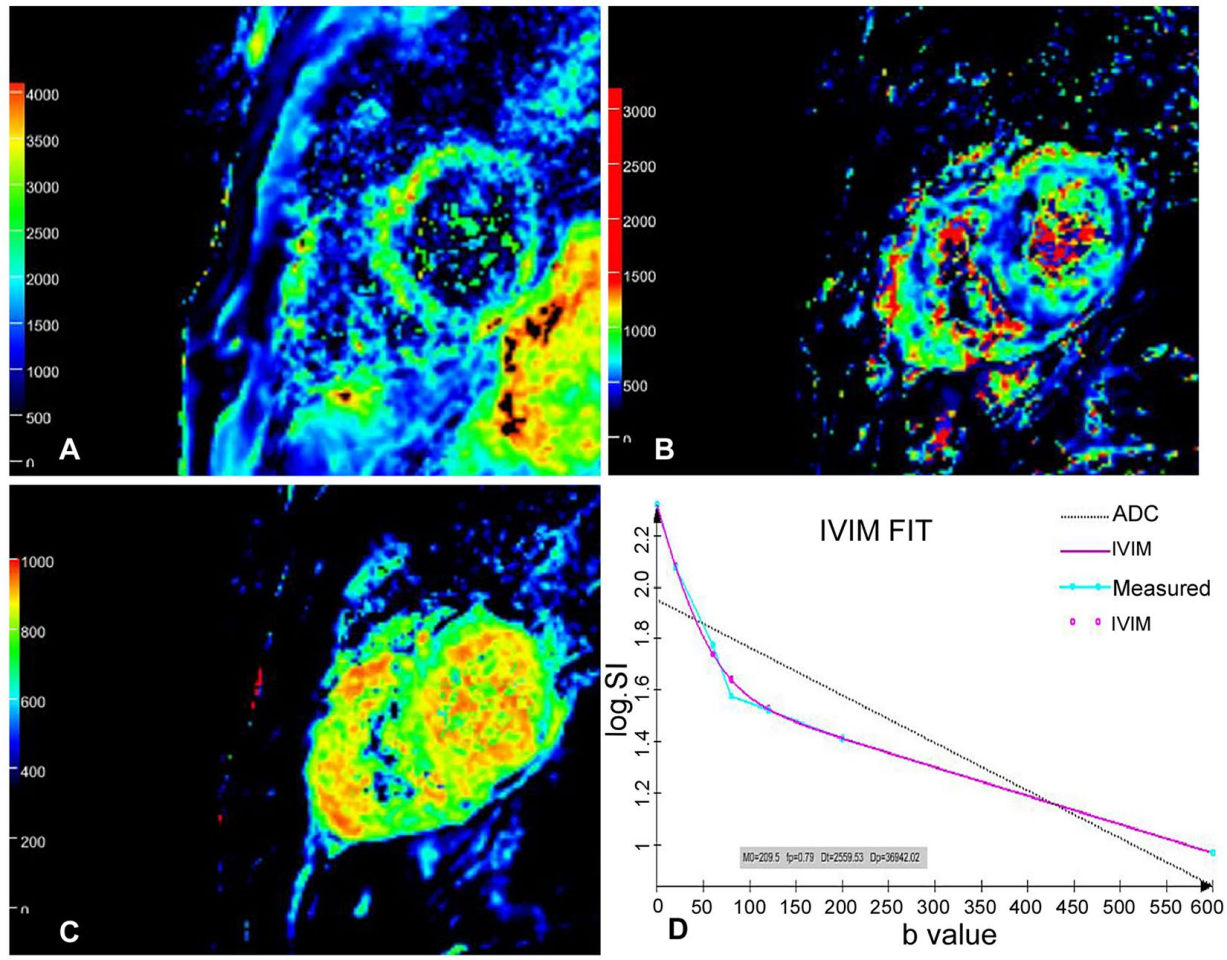
Pseudo-color images of D (**A**), D* (**B**) and ***f*** (**C**) value in IVIM-DWI images in the same 51-year-old healthy volunteer as in Fig. 3. The D, D* and ***f*** values of the left ventricular myocardium were uniform. The D, D* and ***f*** values of the interventricular septum were 0.0028 × 10^−3^ mm^2^/s, 0.61 × 10^−3^ mm^2^/s and 0.22, respectively. D value, diffusion coefficient; D* value, perfusion coefficient; ***f*** value, perfusion fraction; IVIM, intravoxel incoherent motion; DWI, diffusion-weighted image. (**D**) The measured signal intensity points are distributed near the fitting curves, which reflect a higher image quality without obvious impact by respiratory movement and heartbeat. Meanwhile, the dual-exponential algorithm demonstrates a better fitting effect than mono-exponential algorithm.

Original DWI images of the left ventricular short axis at seven different b-values were obtained in all the 35 patients with old myocardial infarction and the 12 healthy volunteers. When b = 0 s/mm^2^, the cardiac blood flow signal showed a high intensity and slightly raised signals in the myocardium. With increasing b-values, the cardiac blood flow signal decreased gradually to no signal, and the myocardium signal decreased gradually; the signal intensity of the myocardium was relatively low when the b-value was 600 s/mm^2^. Among the 35 patients with old myocardial infarction, 96 LGE-positive segments were found among 560 segments scanned in these 35 patients (16 segments for each patient). With the increase in the b-value, there was an increased trend in the signals of infarcted myocardium and a decreased trend in those of the surrounding normal tissues, resulting in a significant contrast (Figs. 1, 2, 3 and 4). Furthermore, pseudo-colour images of the D, D* and *f* values were created, showing the infarcted and normal myocardium of the two groups (Figs. 2 and 4) from the results of the parameters shown in Table 1.

**Table 1:** Parameters of D, D* and ***f*** values between normal and infarcted myocardium (median, inter-quartile range).

Parameters	Normal myocardium		Infarcted myocardium		*Z*	*P*
	Median	Inter-quartile range	Median	Inter-quartile range		
D (×10^−3^ mm^2^/s)	0.0032	0.0016	0.0012	0.0007	−5.028	0.000
D* (×10^−3^ mm^2^/s)	0.5500	0.1650	0.8500	0.1250	−5.582	0.000
*f* (%)	0.2300	0.1475	0.1200	0.0700	−5.257	0.000

Note: D, diffusion coefficient; D*, perfusion coefficient; ***f***, perfusion fraction.

### Dynamic change of IVIM-DWI-associated parameters (D, D* and *f* values)

**Figure 5:**
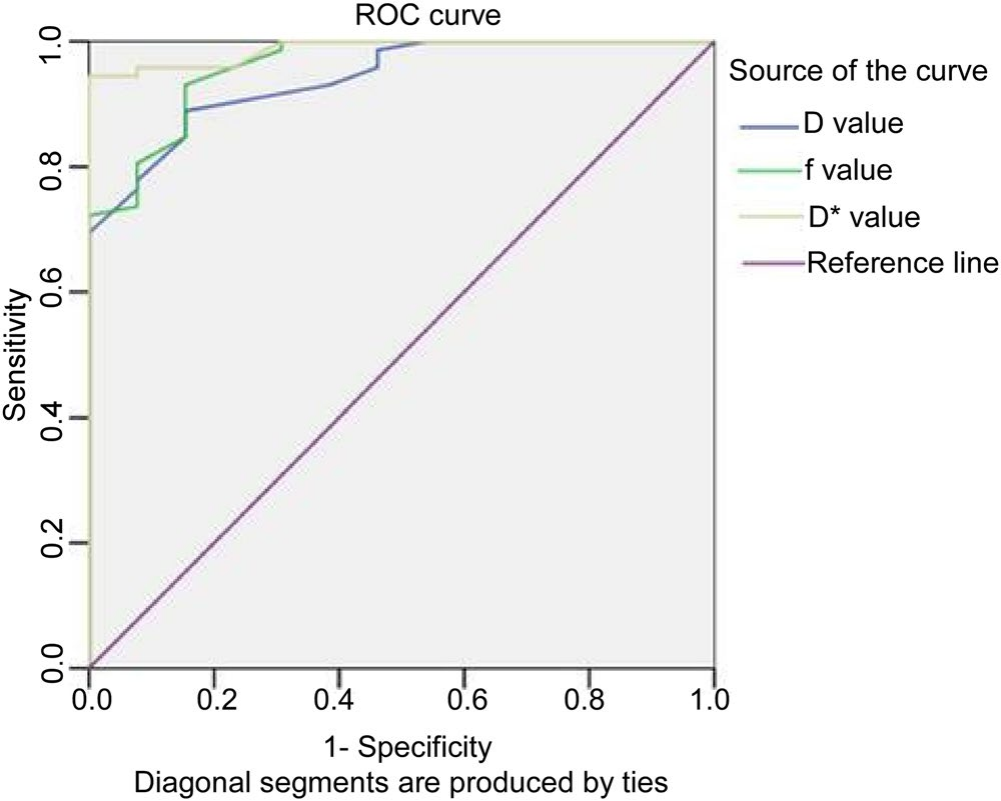
Comparison of the area under the receiver operating characteristics (ROC) curve of the intravoxel incoherent motion-diffusion-weighted imaging (IVIM-DWI) parameters of D, D* and *f* value between normal and infarcted groups. The area under the curve (AUC) was 0.939, 0.988 and 0.959, for D, D* and ***f*** value, respectively. D value, diffusion coefficient; D* value, perfusion coefficient; ***f*** value, perfusion fraction.

There were significant differences in the mean D, D* and *f* values between the 35 patients with infarcted myocardium and the 12 individuals with normal myocardium (Z = −5.028, −5.582 and −5.257, *P* < 0.05). Furthermore, the area under the curves (AUC) in ROC analysis of the D, D* and *f* values in the two groups were 0.939, 0.988 and 0.959, respectively (Table 2). The sensitivity and specificity in the healthy volunteer group and the myocardial infarction group were 84.6% and 88.9% for the D, 100% and 94.4% for D* and 84.6% and 93.1% for *f* values, respectively (Fig. 5). Power analysis with the G* Power 3.1.9.2 version (Franz Faul, Uni Kiel, Germany) showed that if the statistical power was 80%-90% with the α = 0.05, the total sample size would be 27–36 for one tail and 34–44 for two tails.

**Table 2:** Receiver operating characteristics (ROC) curve analysis.

Variables	Cut-off Value	Senstivity	Specificity	AUC
D value	0.00155	84.6%	88.9%	0.939
f value	0.165	84.6%	93.1%	0.959
D* value	0.74	100%	94.4%	0.988

## Discussion

In the calculation of apparent diffusion coefficient (ADC) value, a least square method is used to fit the value because there is a linear relationship between b values and the logarithmically converted signal intensity. An equation of mean square difference (MSE) can be calculated using all the acquired b values. After the equation is established, the first derivative MSE equation is calculated, and then, the b value that can help getting the minimal MSE is calculated. For the calculation of D, D* and f values, a similar method was used according to a dual-exponential algorithm. As in other IVIM studies, a more numerically stable “segmented” analysis procedure was performed. Because D* is significantly greater than D, when the b-value is significantly greater than 1/D*, the influence of the pseudo-diffusion term on the signal decay is quite small. Thus, in this higher b-value regime, the algorithm can be simplified into a mono-exponential equation, whereby D and f can be estimated. With D and f values calculated previously, D *values can be calculated by using a partially constrained nonlinear fit of the entire dataset according to the dual-exponential algorithm. The DWI is an important examination method in functional MRI at present. However, the ADC value, calculated using the traditional single-exponential model, fails to consider the signal loss resulting from water molecular displacement caused by the intra-tissue microcirculation perfusion, thereby affecting the accuracy of tissue ADC value evaluation. Notably, the IVIM double-exponential model can calculate the D value (tissue diffusion movement), D* value (change of diffusion coefficient caused by microcirculation perfusion) and *f* value (perfusion fraction), by acquiring DWI images at several b-values (>4). Thus, such a model can be used to evaluate tissue diffusion and microcirculation perfusion simultaneously, indicating the great potential of this technique to accurately evaluate the myocardial perfusion and water molecular diffusion^4–8^. Additionally, the IVIM-DWI can provide micro-perfusion information, while avoiding the risks of contrast agent allergy and gadolinium-related renal fibrosis, as it avoids the need to inject the contrast agents.

The infarcted myocardium of anterior and inferior segments was displayed as high signal on DWI (Fig. 1). In order to decrease measurement errors, regions of interest (ROI) were firstly drawn on high b value images, and then, the relevant ROI was produced in the pseudo-color images automatically with recording of the relevant value of D, D* and f. Through analysis of these values in infarcted and normal myocardium, fibrosis of myocardium was evaluated through changes of pseudo-color. Using the IVIM dual-exponential algorithm for measurement of functional parameters, the D value indicates the diffusion status of water molecules inside the myocardium, and the infarcted myocardium showed decreased D values correlating to the LGE-positivity. Data acquisition in IVIM-DWI currently relies generally on the echo-planar imaging (EPI) technology in clinical practice. Nevertheless, the complexity of cardiac anatomy and physiological movement affects the image quality in the DWI, which in turn affects the accuracy of IVIM-DWI parameters. In this experiment, iShim sequence was used for the IVIM-DWI imaging, which is an improvement in the traditional EPI sequence compared with the traditional DWI^7^. In comparison with the conventional single-shot EPI sequencing, the advantages of the iShim sequencing lie in the facts that: (1) corresponding b-values can be selected reasonably according to the requirements of the experiment, especially when the b-values are small, so as to improve the fitting accuracy of the D* value^8^; (2) it can reduce image distortion; the iShim sequence scans a B0-Map before scanning each slice, and the signal changes of the B0-Map are used to correct the distortion of EPI, thereby contributing to the improvement in the distortion caused by the heterogeneity of the main magnetic field; (3) it can exert a better fat-suppression effect; specifically, the sequence can achieve a more thorough suppression of fatty tissue signals, by improving the accuracy of fat-selective pulses and using a slice-selection gradient-reversal fat-suppression technique; (4) it optimises the image acquisition mode, allows higher acquisition times, increases the signal-to-noise ratio and further improves the accuracy of parameter fitting. The setting of the b-value is also quite important in improving the accuracy of the parameter values. A lower b-value is more sensitive to perfusion of microcirculation, which, however, is prone to producing a ‘T2 penetration’ effect. Higher b-values may express a higher sensitivity to the diffusion of water molecules, but are also accompanied by a higher risk of image anatomical deformation and distortion that may affect the accuracy of the measurement^9–11^. In addition, in view of the continuous beating of the heart and the respiratory movement of the chest, breathing navigation technology and ECG-gated technology also play critical roles in improving the image quality. In this study, six low b-values, ranging 0–200 s/mm^2^, and one high b-value of 600 s/mm^2^ was selected for short axis DWI, in combination with the application of reduced field-of-view and local shimming. Meanwhile, respiratory navigation and ECG gating techniques were also used to improve the quality of the scanned images. Consequently, images of 35 patients with old myocardial infarction and 12 healthy volunteers were screened and included for analysis, without significant loss of myocardial signal and a clear display of myocardial contour.

In 2003, Callot *et al*.^12^ confirmed the feasibility of IVIM imaging for the first time by using IVIM technology in the canine heart. Delattre *et al*.^13^ used an IVIM model of *in vivo* cardiac MRI and measured the parameters of IVIM in the human heart for the first time. In addition, Rapacchi *et al*.^14^ measured IVIM imaging of 10 normal human hearts by the EPI technology. In view of the results of these studies, all the experiments indicated that the D, D* and *f* values of 17-segment MRI of the left ventricle were not completely equal, despite the existence of some differences in the parameters, similar to the results of the present study. In our study, normal myocardial parameters were obtained in 12 healthy volunteers. D values of the anterior and inferior left ventricular septum were larger than those of the free wall myocardium, while converse results were found for the D* and *f* values, indicating significant differences among the parameters. The results suggested differences in the myocardial perfusion blood flow of the anterior descending branch, the right coronary artery and the circumflex branch. The 17-segment model is currently widely applied worldwide for left ventricle segmentation^9,11,15^. Different segments of the left ventricle are supplied by the anterior descending branch, the circumflex branch and the right coronary artery. Because most of the Chinese population have the right dominant type of coronary artery, the anterior wall and anterior ventricular septum of the left ventricle are thus mainly supplied by the anterior descending branch, the inferior wall and inferior septum are mostly supplied by the right coronary artery, and the lateral wall is predominantly supplied by the circumflex branch. The blood flow of the anterior descending branch and the right coronary artery is generally larger than that of the left circumflex artery. Thus, the results of this experiment were reasonable to certain extent. Power analysis showed that the total sample size in our study is enough to draw the conclusion with well over 80% power. In a study conducted by Kociemba *et al*.^16^, a 1.5 T MR scanner was used, and DWI sequences with b-values of 0, 50, 100 and 200 s/mm^2^ were used for cardiac scanning. The results showed that the DWI possessed high sensitivity and specificity in the detection of acute myocardial infarction. Furthermore, in a study by Laissy *et al*.^17^, the ADC value in the healthy volunteers was higher than in patients with myocardial infarction in different periods, when the b-value of a 1.5 T MR scanner was approximately 300 s/mm^2^, consistent with the results demonstrated in our study. Our study indicated that the D and *f* values of infarcted myocardium in 35 patients with old myocardial infarction were significantly lower than those in the 12 healthy volunteers and that the D* value was significantly higher than that of the normal myocardium. We speculated that this was related to decreased blood perfusion caused by ischaemic necrosis and infarcted myocardium with decreased blood flow. Moreover, the AUCs of the D, D* and *f* values were 0.939, 0.988 and 0.959, respectively, with no significant difference in the diagnostic efficiency. Furthermore, the infarcted myocardium of 35 patients with old myocardial infarction showed high signal on DWI in a bi-exponential model. When b = 0 s/mm^2^, the signal intensity of the blood pool was high, which easily affected the display of the subendocardial infarcted myocardium^18–22^. With an increasing b-value, the intracardiac blood pool showed low signals, and thus, a higher b-value might induce a more obvious contrast between infarcted and normal myocardium, aiding the diagnosis of infarcted myocardium.

Quantitative detection of fibrosis in old myocardial infarction plays an important role in evaluating the degree of left ventricular dysfunction and progression to heart failure^23–26^. At present, delayed enhancement is the gold standard for the diagnosis of fibrosis in old myocardial infarction, which, however, possesses deficiencies due to the requirement for injection of contrast agent, long scanning time and no quantitative indicators. However, the IVIM-DWI technology can display and quantitatively detect myocardial fibrous scars without the need for gadolinium-containing contrast agents, with corresponding precision, confirmed experimentally^27–29^. The mechanism leading to decreased D and f values but increased D* value in the fibrosis of myocardium in IVIM of MRI may include the following several aspects. As increase of the fibrous connective tissue component in the fibrous myocardium, especially increase of the collagen fiber composition, the Brownian motion of water molecules of the fibrous myocardium is damaged, resulting in decrease of the D and f values. Fibrosis of the myocardium is accompanied by degeneration of myocardial cells to cause increase of the tissue space of the myocardial collagen fibers, leading to decreased perfusion and subsequent increase of the D* value. In our study using the IVIM dual-exponential algorithm for imaging the myocardium with several b values, the D, D* and f values can be simultaneously obtained to reflect the water movement, dispersion and blood microcirculation perfusion state. As increase of the b values, the myocardial signal begins to attenuate, and the infarcted myocardium is of high signal. The D, D* and f values measured quantitatively with the LGE standard in the normal and infarcted myocardium were analyzed to determine the cut-off value, sensitivity, specificity and AUC, which demonstrated high values of these parameters in diagnosis of myocardial infarction. The results of the current study also supported the hypothesis that IVIM-DWI can diagnose old myocardial infarction more easily, and the parameters of the D, D* and *f* values can quantitatively evaluate the extent and degree of myocardial infarction fibrosis. Therefore, the IVIM-DWI has a certain value in demonstrating the blood perfusion and water molecular diffusion of old myocardial infarction.

This study has some limitations including a limited cohort, retrospective nature, non-randomization, single center study and only Chinese ethnicity involved. Future studies will have to solve the problems for better outcomes.

In conclusion, the IVIM-derived parameters (D, D* and *f*) obtained using the iShim DWI technique showed high capacity in diagnosing old myocardial infarction and myocardial fibrosis by providing diffusion and perfusion information, which may have great importance in future clinical practice.
